# Long-term radiographic and periodontal evaluations of the bone-grafted alveolar cleft region in young adults born with a UCLP

**DOI:** 10.1093/ejo/cjad064

**Published:** 2023-11-13

**Authors:** Mathias Lemberger, Petra Peterson, Anna Andlin Sobocki, Hedieh Setayesh, Agneta Karsten

**Affiliations:** Division of Orthodontics and Pediatric Dentistry, Department of Dental Medicine, Karolinska Institutet, Box 4064, 141 04 Huddinge, Sweden; Eastman Institute, Department of Orthodontics, Public Dental Services Stockholm, Box 6031, SE-102 31 Stockholm, Sweden; Department of Molecular Medicine and Surgery, Karolinska Institutet, 171 77 Stockholm, Sweden; Department of Plastic and Reconstructive Surgery, Karolinska University Hospital, SE-171 76 Stockholm, Sweden; Department of Surgical Sciences, Uppsala University and Akademiska hospital, 751 85 Uppsala, Sweden; Division of Orthodontics and Pediatric Dentistry, Department of Dental Medicine, Karolinska Institutet, Box 4064, 141 04 Huddinge, Sweden; Division of Orthodontics and Pediatric Dentistry, Department of Dental Medicine, Karolinska Institutet, Box 4064, 141 04 Huddinge, Sweden

**Keywords:** alveolar bone grafting, bone graft, long-term, periodontal, radiographic, cleft lip, cleft palate

## Abstract

**Background:**

Studies addressing the periodontal health of the teeth surrounding the bone-grafted cleft in patients born with unilateral cleft lip and palate disagree on whether periodontal health is compromised.

**Objectives:**

To determine periodontal health differences between the cleft and the non-cleft sides nearly a decade after secondary alveolar bone grafting.

**Methods:**

This prospective, controlled (split-mouth design) study comprised an intraoral apical radiographic and a periodontal examination of 40 consecutive patients from one centre (*n* = 26 males) who had undergone bone grafting at mean age of 10.2 years (±1.6). Probing pocket depth, gingival index, gingival recession, and radiographic bone support were assessed.

**Results:**

No significant difference occurred in probing pocket depth between teeth at cleft and non-cleft sites (OR 1.8, *P* = .488). Gingival recession was present at 6.6% of all examined sites on the cleft side and at 1.7% on the non-cleft side (OR 17.3, *P* < .001). Gingival recession occurred most often on the buccal and disto-buccal surfaces of the central incisor on the cleft side. The gingival index was significantly higher on the cleft side (OR 8.0, *P* < .001). The Bergland index was I or II in most patients (87%).

**Limitations:**

Recruitment of eligible patients was lengthy.

**Conclusion:**

The teeth on the cleft side had high levels of gingival inflammation. Few pathological gingival pockets, however, were found. Shallow gingival recessions frequently occurred around the central incisor on the cleft side. Teeth in the bone-grafted cleft region generally had good bone support.

## Introduction

Management of alveolar clefts usually involves autologous bone-graft harvesting to fill the cleft in order to unify the maxillary segments and provide bone support for the erupting secondary teeth in the cleft zone [1–3]. Other goals include closing any oronasal fistulas and, in addition, correcting the alveolar contour and strengthening alar base support to improve dental and facial aesthetics. Bone grafting also supports final prosthetic restorations of the teeth [4–7].

Most cleft centres perform alveolar bone grafting (ABG) in the mixed dentition (secondary ABG) as maxillary growth seems to be less disturbed than when ABG is performed in the primary dentition (primary ABG [8–10]). Clinicians have traditionally evaluated bone grafting on conventional intra-oral radiographs. Several cleft centres now prefer to use cone beam computed tomography (CBCT) as it permits three-dimensional (3D) assessment of the bone-grafted cleft, even though it delivers more radiation to the patient [11–14].

Several grading systems have been developed for quantitatively evaluating the bone-grafted alveolar cleft on conventional intraoral radiographs, including the Bergland index (BI; interdental vertical bone height; 2), the Kindelan scale (degree of bony fill in the cleft area [15];), and the Witherow scale (position and distribution of the bone graft within the cleft [16];). Toscano et al. [17] found that an 87% concordance between the BI and the Witherow scales, indicating that they were comparable. Studies have reported long-term radiographic outcomes for secondary ABG of between 58% and 98% [2, 17–23].

Some authors find no difference between using the BI on occlusal radiographs compared with the equivalent index on CBCT images; hence, conventional occlusal radiographs seem to provide an adequate image for evaluating postoperative vertical bone height in clinical follow-ups [24]. Meazzini et al. [25] reported a 95% success rate of alveolar bone healing as measured on panoramic radiographs (BI of I and II) of patients with uni- and bilateral cleft lip and palate who had undergone early secondary gingivoalveoloplasty. Long-term results seem to show that the Milan surgical protocol also allows adequate ossification of the alveolar and nasal region [26]. However, long-term follow-ups of patients treated with primary periosteoplasty only or in combination with later secondary ABG found bone formation to be inferior compared with patients treated with secondary ABG only [27, 28].

It is also known that the outcome of secondary ABG improves if the bone graft is performed just before the canine erupts [29–35]. Autologous bone harvested from the iliac crest, mandibula, fibula, or calvarium is the most common source of bone for grafting [36–38].

The most important goal of bone grafting the alveolar cleft is to create an osseous environment for erupting teeth. However, post-surgical periodontal status varies among patients, and the limited literature disagrees on whether the periodontal health of the teeth within the alveolar cleft is compromised. Some studies claim that gingival and periodontal pathologies are not affected by teeth close to the cleft [39–41]. In contrast, other studies report that teeth surrounding the cleft are more susceptible to periodontal disease and gingival recession [42–45]. Improving dental status regarding tooth position and oral hygiene improves the long-term bone graft outcome. However, vertical bone height still tends to decrease with time [46].

The literature currently has an evidence gap concerning long-term results of secondary ABG that includes a sparsity of radiographic evaluations and assessments of clinical periodontal variables. Hence, the aim of this prospective follow-up study was to evaluate the long-term outcome of secondary ABG in young adults born with unilateral cleft lip and palate (UCLP) using a split-mouth design with the outcome variables periodontal health and bone support of the rehabilitated reconstructed alveolar cleft.

## Subjects and methods

### Study population

A consecutive series of 73 children born with UCLP between 1992 and 2000 were recruited at their last, post-surgical, follow-up examination. The inclusion criteria were surgical treatment (lip repair, palate repair, and bone grafting) according to the standard protocol in the Stockholm cleft centre. Exclusion criteria were associated syndromes. Six patients were excluded because of syndromes and 16, because they had moved. Three patients were excluded because the standard surgical protocol had not been used, and one patient was deceased. Seven patients missed their last follow-up examination and were therefore excluded. After oral and written information about the study, the included patients signed the consent forms. The final study cohort comprised 40 patients (*n* = 14 females).

### Treatment protocol

A lip repair according to Tennison–Randall [47] was done at a mean age of 4.6 months (±1.4). The palatal repair was done according to the minimal incision technique (MIT) at a mean age of 13.8 months (±4.4). Mendoza et al. [48] first described the MIT. Karsten et al. describe the MIT in detail [49].

Bone grafting was performed at a mean age of 10.2 years (±1.58) according to Boyne and Sands [50] with autologous bone harvested from the iliac crest. Timing of bone grafting was based on the degree of root development of the lateral incisor or the canine adjacent to the cleft and the proximity of the erupting tooth to the cleft. When present, the lateral incisor was preserved.

### Clinical examination

**Figure 1.**
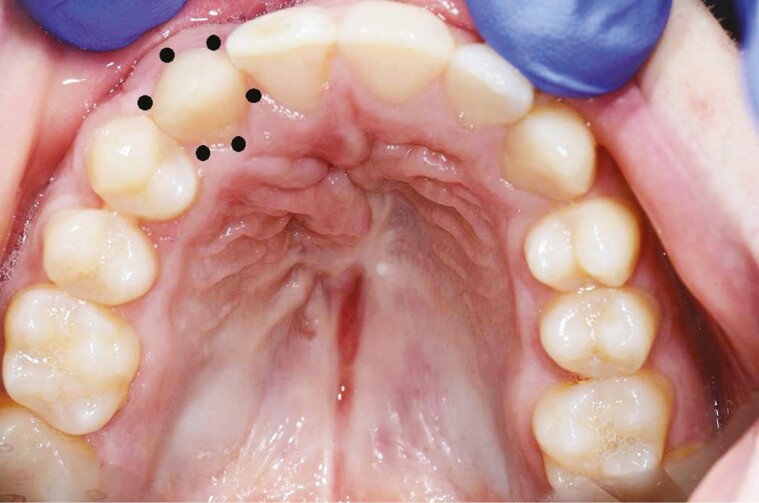
The six sites (b = buccal, db = disto-buccal, dl = disto-lingual, l = lingual, mb = mesio-buccal, and ml = mesio-lingual) of periodontal pocket depth and bleeding on probing measurements.

**Figure 2.**
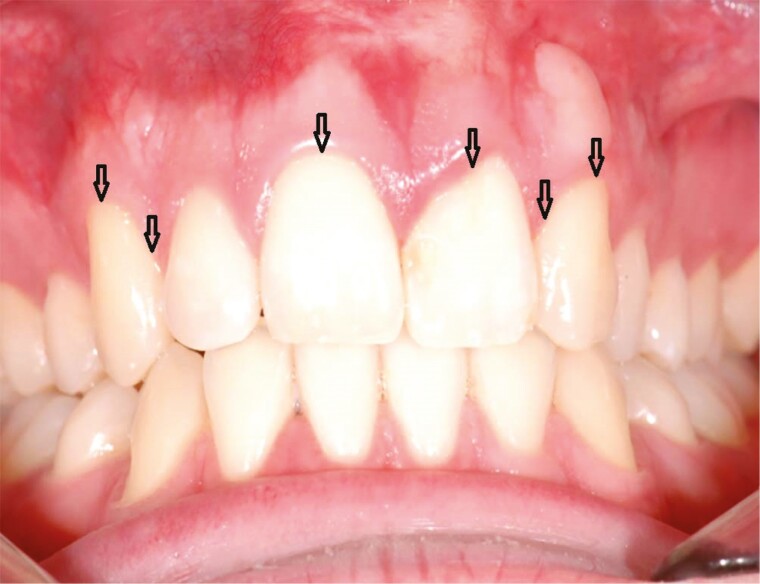
The split-mouth design where the teeth on the cleft side (central and lateral incisors and canine, when present) were compared with the same (control) teeth on the contralateral, non-cleft side.

All eligible patients were invited to a clinical examination. Mean age at the examination was 19.9 years (± 1.2). In all, 204 maxillary teeth were examined with a mean of 5.1 teeth per patient (Table 1). Clinical measurements with a periodontal probe (UNC 15; Hu-Friedy, Chicago, IL) were taken at six sites for each examined tooth (Fig. 1). In this study, the non-cleft side served as the control (split-mouth design). Each examined site on the cleft side was compared with the corresponding site on the contralateral side (Fig. 2). Periodontal probing depths and gingival recessions were measured in mm. The examination assessed the presence or absence of gingival inflammation according to the Löe and Silness gingival index (GI) with four possible scores: 0 = normal gingiva; 1 = mild inflammation, no bleeding on probing (BOP); 2 = moderate inflammation, BOP; and 3 = severe inflammation, BOP, and occasionally spontaneous bleeding [51]. One author (M.L.) made all measurements.

**Table 1.** The examined teeth (central and lateral incisors and canine on either side of the operated cleft, when present) nearly a decade after alveolar bone grafting in the study cohort of patients (*n* = 40) born with UCLP.

Tooth	Cleft side (*n*)	Non-cleft side (*n*)	Total (*n*)
Central incisor	36	40	76
Lateral incisor	12	36	48
Canine	40	40	80
Total	88	116	204

### Radiographic assessment

Mean age at the radiographic examination was 19.5 years (±1.2). Thirty-nine apical radiographs were assessed using the BI. The apical radiograph for one patient was missing. The BI classifies vertical bone height in four grades. Grade I is defined as approximately normal bone height; grade II, bone height at least ¾ of normal height; grade III, bone height less than ¾ of normal height; and grade IV, which implies that no continuous bone bridge crossing the cleft can be identified [2].

### Statistical analysis

R Core Team (2021, Version 4.1.1; R Foundation for Statistical Computing, Vienna, Austria) was used in the statistical analysis. The differences between the cleft and non-cleft sides in gingivitis, gingival recession, and periodontal probing depth were assessed using McNemar’s test for paired samples. Risks of developing gingivitis, recessions, and pathological periodontal pockets on the cleft side compared to the non-cleft side were calculated in generalized linear mixed-effects (GLME) models with the odds ratio (OR). The GLME model was also used to determine the consequences of the periodontal status of a missing lateral incisor on the cleft side. In addition, this model was used to test for significant differences in the ORs of gingival recessions between space opening and space closure with the presence of a lateral incisor on the cleft side. A power analysis for the BOP variable (alfa = 0.05, beta = 0.2, standard deviation = 30%, and mean difference between the cleft and non-cleft sides = 15%) estimated a sample size of 34 patients would be necessary to detect a significant difference.

### Reproducibility of recording

One orthodontist (M.L.) and one general dentist (H.S.) analysed all intra-oral apical radiographs twice. Intra- and interobserver reliability for the BI was calculated with Cohen’s kappa.

### Ethical approval

The Regional Ethical Review Board in Stockholm approved the present study (Dnr [daybook no.] 2008/502-31-2 and Dnr 2016/1663-32).

## Results

### Sample characteristics

The study cohort comprised 40 patients (14 females). The cleft in 26 patients (65%) was on the left side and in 14 (35%), on the right side. Twelve (30%) of the patients had a lateral incisor on the cleft side. All patients had received orthodontic treatment and five were still wearing braces. In the 21 (52.5%) patients with a missing lateral incisor, the gap had been closed with orthodontic treatment. Five of the patients with a missing lateral had received a resin-bonded fixed dental prosthesis to replace the missing tooth; one patient, a removable prosthesis; and one, a dental implant (Table 2).

**Table 2.** Patient characteristics in the study cohort (*n* = 40; female: *n* = 14, 35%) of patients who were born with UCLP (right side: *n* = 14, 35%; left side: *n* = 26, 65%) and had undergone alveolar bone grafting approximately 10 years earlier.

	No (%)	Yes (%)
Lateral incisor on the cleft side	28 (70)	12 (30)
Resin-bonded fixed dental prosthesis	35 (87.5)	5 (12.5)
Orthodontic space closure	21 (52.5)	19 (47.5)
Dental implant	39 (97.5)	1 (2.5)
Prosthetic tooth	39 (97.5)	1 (2.5)
Bonded retainer maxilla	24 (60.0)	16 (40.0)
Dental braces	35 (87.5)	5 (12.5)
Orthodontic space opening	31 (77.5)	9 (22.5)

### Periodontal probing depths

The median probing depth on both sides was 2 mm (range: cleft side, 1–5 mm; non-cleft side, 1–4 mm). Pathological pockets of 4 mm or more occurred in 0.8% of the examined sites on the cleft side and 0.4% on the non-cleft side. The differences between cleft and non-cleft sites were not significant (OR 1.76, *P* = .488).

### Gingival index

**Figure 3.**
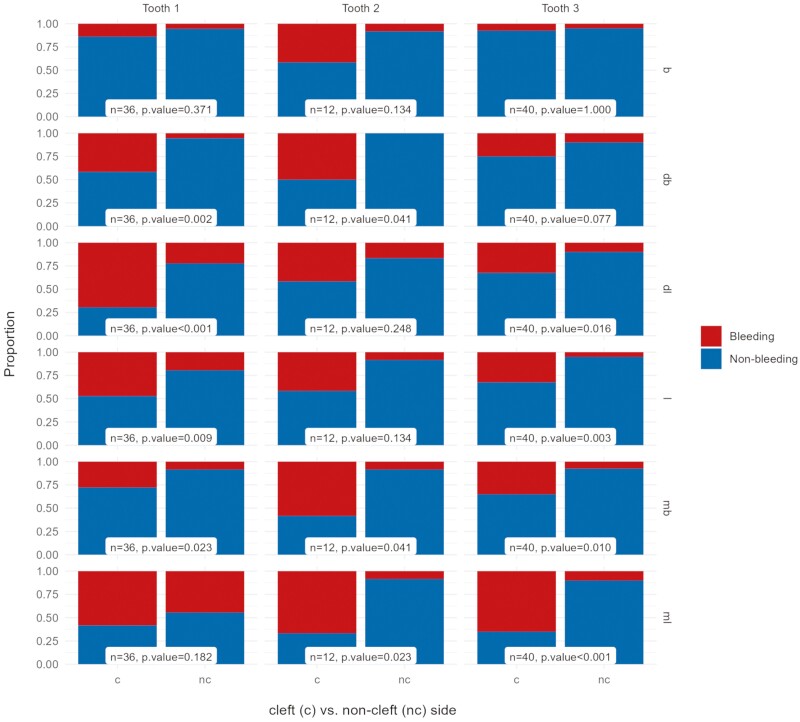
Proportion of tooth surfaces (b = buccal, db = disto-buccal, dl = disto-lingual, l = lingual, mb = mesio-buccal, ml = mesio-lingual) with and without bleeding per tooth (1, central incisor; 2, lateral incisor; 3, canine) on the cleft (experimental) and on the non-cleft (control) side.

A GI of 2 or 3, which indicates BOP, occurred at 39% of the examined sites on the cleft side and at 14% on the non-cleft side, a significant difference (OR 8.02, *P* < .001). More specifically, BOP was significantly higher on the central incisor on the cleft side compared to the non-cleft side disto-buccally (*P* = .002), disto-lingually (*P* < .001), and lingually (*P* = .003). BOP was also significantly higher on the cleft-side canine lingually (*P* = .003) and mesio-lingually (*P* < .001; Figure 3). Patients with a missing lateral incisor on the cleft side did not have a higher risk of BOP compared to patients who had a natural lateral incisor on the cleft side (*P* = .683). The five patients with a BI of III or IV did not have higher levels of gingivitis compared to the patients with a BI of I or II (*P* = .996).

### Gingival recession

**Figure 4.**
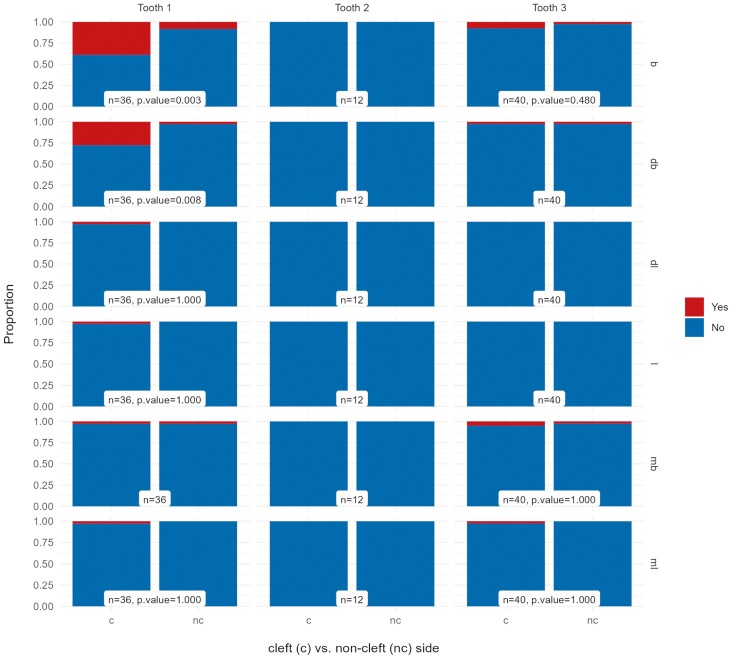
Proportion of tooth surfaces (b = buccal, db = disto-buccal, dl = disto-lingual, l = lingual, mb = mesio-buccal, ml = mesio-lingual) with and without gingival recession per tooth (1, central incisor; 2, lateral incisor; 3, canine) on the cleft (experimental) and on the non-cleft (control) side.

Gingival recession was observed at 6.6% (*n* = 35) of all the examined sites on the cleft side compared to 1.7% (*n* = 12) on the non-cleft side (OR 17.3, *P* < .001) and occurred most often on the buccal and disto-buccal surfaces of the central incisor on the cleft side. Thirty-nine per cent (*n* = 14) of the central incisors on the cleft side presented with gingival recession on the buccal and 28% (*n* = 10) on the disto-buccal side (Fig. 4). Gingival recessions were found in 80% of patients with a BI of III or IV compared to only 41% of patients with a BI of I or II. The ORs for gingival recession between the presence of a lateral incisor on the cleft side and orthodontic space opening (*P* = .286) or orthodontic space closure (*P* = .354) did not differ significantly.

### Bergland index

**Figure 5.**
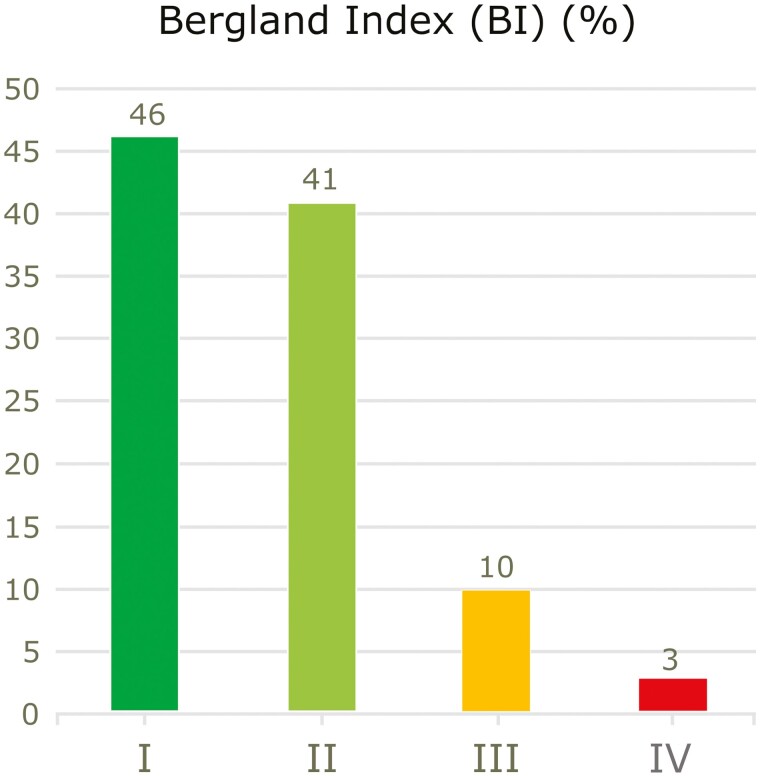
Proportion of patients (*n* = 39) with normal or less than normal alveolar bone height nearly a decade after alveolar bone grafting, measured with the Bergland index (BI) on intraoral apical radiographs (normal or almost normal: BI I and II; less than normal or no bone: BI III and BI IV).

For the two raters of the BI, intra-observer (*κ* = 0.864, *κ* = 0.898) and inter-observer (*κ* = 0.898, *κ* = 0.864) agreements were strong. A BI of I or II was seen in 87% and a BI of III or IV in 13% of the patients (Fig. 5). No patient with a BI of III or IV had a lateral incisor on the cleft side. However, as assessed with the BI, the bone graft outcomes of patients with and patients without a lateral incisor did not differ significantly (*P* = .299).

## Discussion

This prospective, controlled, long-term follow-up study assessed the radiographic outcome of alveolar bone grafting in individuals born with UCLP and their clinical periodontal health nearly 10 years after surgery. The split-mouth study design was used to control for confounding factors. The study group was homogenous for patient age and diagnosis. Treatment of all patients followed the same protocol, and the same investigator made all clinical measurements (M.L.). One potential confounding factor was that five patients were wearing braces at the time of the follow-up examination. However, the braces had been placed on both sides of the dental arch. In the split-mouth design, the effect being studied is controlled. Thus, every patient acted as their own control: the non-operated side was the control for the operated, cleft side, and the study design was able to control for the presence of braces in the patients who were still wearing them.

The main finding of this study was the higher level of gingivitis on the cleft side compared to the non-cleft side: disto-buccally, disto-lingually, and lingually on the central incisor; and lingually and mesio-lingually on the canine. Possibly, gingival inflammation occurs more frequently on the surfaces facing the cleft. The Passinato Gheller et al. [44] study reported higher levels of gingivitis in patients born with a cleft lip and/or palate (age range 6–18 years of age), compared to a control group. However, they did not report gingivitis on the tooth surface level. Dewinter et al. [52] found no signs of increased gingival inflammation in a split-mouth design study on 75 patients with UCLP. However, the age range among the patients varied between 8 and 20 years; hence, the study group was rather heterogeneous. Prayitno et al. [53] considered gingivitis to be a poor predictor of periodontitis in subjects younger than 30 years. Nevertheless, gingivitis and periodontitis are believed to be a continuum of the same inflammatory disease [54], and while not all patients with gingivitis will experience a progression to periodontitis, management of gingivitis is a primary prevention strategy for periodontitis and a secondary prevention strategy for recurrent periodontitis [55].

An apically positioned marginal gingiva that leaves the root surface exposed to the oral environment is the main characteristic of a gingival recession. Insufficient alveolar bone over the root surface might be a risk factor for developing recession. We found gingival recessions more frequently on the cleft side and most often on the buccal and disto-buccal surfaces of the central incisor. Gingival recession might be regarded as an aesthetic problem but is associated with compromised plaque control and can lead to dentin hypersensitivity [56, 57]. In the present cohort, 1% of the examined sites on the cleft side had a major recession of 5 mm or more. If the recession is major, the risk of tooth loss due to tissue loss might be higher. On the non-cleft side, no recession of 5 mm or more was found. Gingival recession was found in 80% of the patients with a BI of III or IV compared in 41% of patients with a BI of I or II. Hence, a poor bone grafting outcome that fails to provide enough bone in the cleft zone might be a risk factor for the development of gingival recessions. Additionally, orthodontic treatment and the mechanical trauma of tooth brushing in combination with insufficient bone support could also be a risk factor for apical migration of the marginal gingiva, causing gingival recessions. In our split-mouth design study, however, the presence of braces was the same for both sides of the dental arch.

According to the BI, 87% of the patients in the present study had normal or almost normal septal bone height in the bone-grafted cleft zone at 19 years of age. This finding supports the treatment strategy of performing secondary bone grafting in the mixed dentition and is in accordance with reports from other cleft centres. However, the Iino et al. [58] study found that intraoral radiographs may overestimate around 40% of bone graft outcomes. In contrast, Jabbari et al. [23] found no difference when the alveolar bone height was rated on occlusal radiographs with the BI or on CBCT images with an equivalent index.

The Arctander et al. [59] study examined 18 consecutive individuals born with UCLP at least 20 years after they had undergone secondary bone grafting at ages 7–11 years. Although bone mass was significantly lower on the grafted than on the non-grafted side when examined on computed tomography images, overall functional results were considered satisfactory. Yet, the Americleft project [60] concluded that although the Standardized Way to Assess Grafts scale is useful for inter-centre outcome studies that compare average, expected results from differing protocols and surgical skills, two-dimensional images have limited use in clinical treatment planning for individual cases.

In the present study, few pathological periodontal pockets of 4 mm or more were found. This indicates no ongoing periodontitis. Still, the cleft side had significantly higher BOP scores than the non-cleft side. The highest BOP scores occurred at sites adjacent to the cleft, on the central incisors and canines. For most patients, the level of bone height was satisfactory, and patients with a BI of III or IV did not have higher levels of gingivitis than those with a BI of I or II. Neglecting to brush properly on the cleft side could explain the increased levels of gingivitis there. It has been shown that children with cleft lip and/or palate have more caries in the primary dentition than age-matched non-cleft controls, but the difference did not occur in the mixed, young permanent dentition [61], which is somewhat contradictory. The same study on children born with cleft lip and/or palate found enamel defects to be more frequent, especially in the anterior permanent teeth. Pegelow et al. [62] also found that more than half of the children with cleft lip and/or palate suffered from hypoplasia and enamel opacities, which are known risk factors for plaque accumulation and gingivitis. Other local factors that might have had a bearing on the periodontal status on the operated side could be that scared tissue is prone to bacterial retention and a shorter sulcus and lip-strain make brushing more difficult. The status of dental alignment, previous restorations, and right or left-hand brushing may also affect periodontal status. Those local factors are interesting, but they were not the focus of the present study and are topics for future research.

Hence, the periodontal outcome is multifactorial. It is most probably affected by the total cleft situation, including the anatomy of the soft and hard tissues as well as the rehabilitation process. Regardless, patients with a bone-grafted cleft need more information on the long-term risks of gingivitis and help with optimal oral hygiene. Gingivitis can lead to periodontitis, with tooth loss as well as general health problems such as cardiovascular and valvular heart disease [63]. Thus, patients with a bone-grafted cleft require regular monitoring of their oral health to prevent future health problems.

### Limitations

Because patients were recruited from one centre, the time frame needed to include a sufficient number of patients was long. Right or left-hand brushing was not controlled and could have had a bearing on the periodontal status on the operated side.

## Conclusion

Teeth in the bone-grafted cleft region had few pathological periodontal pockets and good bone support in general. A gingival recession of 1–2 mm on the central incisor was a common finding. Gingival inflammation was more frequently observed on the teeth surrounding the cleft. Gingivitis might, in the long run, be a risk factor for developing periodontitis with potential tooth loss and long-term negative effects on general health.

## Data Availability

The datasets analysed in the current study are available from the corresponding author on reasonable request.

## Author’s note

Presented as a poster at the 97th Congress of the European Orthodontic Society, 30 May – 3 June 2022, Limassol, Cyprus.
